# Effect of Non-Alcoholic Fatty Liver Disease on Estimated Glomerular Filtration Rate Could Be Dependent on Age

**DOI:** 10.1371/journal.pone.0130614

**Published:** 2015-06-18

**Authors:** Zhe Shen, Stefan Munker, Fugang Luo, Han Ma, Chaohui Yu, Youming Li

**Affiliations:** 1 Department of Gastroenterology, The First Affiliated Hospital, College of Medicine, Zhejiang University, 310003, Hangzhou, China; 2 Molecular Hepatology-Alcohol Associated Diseases, II. Medical Clinic Faculty of Medicine at Mannheim, University of Heidelberg, 68167, Mannheim, Germany; 3 College of Medicine, Zhejiang University, 310058, Hangzhou, China; Institute of Medical Research A Lanari-IDIM, University of Buenos Aires-National Council of Scientific and Technological Research (CONICET), ARGENTINA

## Abstract

There is a gap between the association of non-alcoholic fatty liver disease (NAFLD) and renal function in an apparently healthy population. This study aims to assess whether NAFLD is associated with estimated glomerular filtration rate (eGFR) levels and to understand early changes of eGFR in NAFLD. A cross-sectional study was performed among apparently healthy persons who underwent general health screening including laboratory assessments and hepatic ultrasonography from January 2013 to December 2013 at the First Affiliated Hospital of Zhejiang University, College of Medicine, China. This study included 1,193 subjects with a mean age of 48 years. Prevalence of NAFLD was 31.3%. Mean eGFR was significantly lower in NAFLD than in controls (107 ± 19 mL/min/1.73 m^2^ *vs*. 113 ± 23 mL/min/1.73 m^2^, *P*<0.001). Correlation analysis between eGFR and NAFLD related risk factors revealed an inverse correlation between eGFR levels and some NAFLD risk factors (all *P*<0.01). All subjects were classified into five phases according to age. Average eGFR levels of NAFLD were lower than controls in three phases for subjects with ≤ 50 years of age (all *P*<0.05), while there were no significant differences on average eGFR levels between NAFLD and controls in two phases for subjects with >50 years of age (Both *P*>0.05). The eGFR level is significantly associated with NAFLD and its risk factors in an apparently healthy population. Effects of NAFLD on eGFR could be dependent on age.

## Introduction

Non-alcoholic fatty liver disease (NAFLD) is a hepatic manifestation of metabolic syndrome. Development of this disease is closely associated with obesity, type 2 diabetes mellitus, dyslipidemia and hypertension; which form a cluster of metabolic disorders that is now identified as a metabolic syndrome [1,2]. Increasing evidence suggests that obesity, diabetes, dyslipidemia and hypertension are all well-known risk factors for chronic kidney disease (CKD) [3–6]. Growing experimental and epidemiological evidence indicates that NAFLD and CKD could share common pathogenic mechanisms and interactions [7–12]. Thus, the association between NAFLD and CKD has become a hot research issue. Moreover, a link between NAFLD and CKD has been shown to be certain in a systematic review and meta-analysis [13].

Mechanisms of NAFLD in developing CKD remains unclear, and disturbances in the renin-angiotensin-aldosterone system could be a risk factor for kidney disease in NAFLD [14]. Whether NAFLD could influence renal function in the early phase or before NAFLD develops CKD remains to be answered. There is a gap between the association of NAFLD and renal function in apparently healthy persons with normal estimated glomerular filtration rate (eGFR) levels and nonhypertensive/nondiabetic diseases. Verifying the influence of NAFLD in kidneys in the early phase would enable us to further understand the relationship of NAFLD and CKD. This study aims to assess whether NAFLD is associated with eGFR levels and to understand the early changes of eGFR in NAFLD.

## Materials and Methods

### Study design and subjects

From January 2013 to December 2013, a population of subjects who underwent health screening including physical examination, laboratory assessments and hepatic ultrasonography at the International Health Care Center, the First Affiliated Hospital of Zhejiang University, College of Medicine, China was evaluated in this study. The style of this annual health screening was the same as a previous study [15], but they were different years of health screening. All subjects voluntarily participated in this study. Subjects with the following criteria were excluded: (i) subjects who drunk excessive alcohol (alcohol consumption per week ≥140 g for men or ≥70 g for women); (ii) subjects who were taking antihypertensive, antidiabetic, lipid-lowering or hypouricemic agents; (iii) subjects with a history of chronic viral hepatitis, autoimmune hepatitis, schistosomiasis japonica, drug-induced liver disease or kidney disease. Finally, a total of 1,193 eligible subjects were enrolled in this study (736 men with a mean age of 48.3 ± 8.4 years and 457 women with a mean age of 47.3 ± 8.4 years). All procedures were approved by the Ethics Committee of the College of Medicine of Zhejiang University. Before the study, each method and its potential risks were explained in detail to each participant, and written informed consent were obtained.

### Physical examination

All subjects were required to fast overnight and invited to have a physical examination in the next morning. After a health habit inventory was recorded, body measurement and blood pressure were performed by a trained physician. Then, body mass index (BMI) was calculated as mass (kg)/height (m2). Waist circumference (WC) was measured with a measuring tape, which was positioned midway between the lowest rib and superior border of the iliac crest as the subject exhaled normally. Three blood pressure measurements were taken with an automated sphygmomanometer on the right arm at a comfortable sitting position after a five-minute rest. The second and third pressure readings were averaged. Systolic blood pressure (SBP) and diastolic blood pressure (DBP) were recorded and used for analysis.

### Laboratory assessments

Peripheral venous blood samples were collected after physical examination and used for the analysis of biochemical values. All serum biochemistries were measured by a Hitachi 7600–110 automatic analyzer (Hitachi Co., Tokyo, Japan). Values included γ-glutamyltransferase (GGT), triglyceride (TG), total cholesterol (TC), high-density lipoprotein cholesterol (HDLc), low-density lipoprotein (LDLc), very low density lipoprotein cholesterol (VLDLc), serum urine acid (SUA), fasting plasma glucose (FPG), glycosylated hemoglobin (HbA1c), and serum creatinine (Scr). Kidney function level was defined by eGFR, which was evaluated by the formula developed and validated in the Modification of Diet in Renal Disease (MDRD) study [16]. The MDRD formula is as follows: eGFR = 186 x SCr-1.154 x age-0.203 x 1.233 (Chinese) x 0.742 (if female).

### Ultrasonographic examination

Hepatic ultrasonography for all subjects was performed by trained ultrasonographists. Ultrasonographic criteria of hepatic steatosis included liver and kidney echo discrepancy, presence of increased liver echogenicity (bright), echo penetration into the deep portion of the liver, and clarity of liver blood vessel structures [17–19].

### Statistical analysis

Statistical analysis was performed with SPSS 13.0 statistical package (SPSS Inc., Chicago, Illinois, USA). Kolmogorov–Smirnov test was used to assess whether continuous data were normally distributed. Continuous variables are presented as mean and standard deviation or median and interquartile range (IQR) as appropriate. Continuous data for different groups were compared using the Student *t*-test or Mann–Whitney *U*-test. Chi-square (χ2) test was used for comparisons of categorical variables. Pearson’s or Spearman’s analysis was used to determine correlations between parameters. *P*<0.05 was considered statistically significant.

## Results

### Subject characteristics

Among the 1,193 included subjects, 373 (31.3%) were diagnosed as NAFLD, and 820 (68.7%) were diagnosed as non-NAFLD. Subject characteristics by NAFLD status are illustrated in Table 1. There was no significant difference on average age between subjects with NAFLD and subjects without NAFLD. Male population, as well as SBP, DBP, BMI, WC, GGT, TG, TC, LDLc, VLDLc, SUA, FPG and HbA1c levels, were significantly higher in the NAFLD group, compared to the control group (All *P*<0.01). Meanwhile, HDLc and eGFR levels were significantly lower in subjects with NAFLD when compared to controls (All *P*<0.01).

**Table 1:** Subject characteristics according to NAFLD status.

Variables	With NAFLD	Without NAFLD	*T* value	*P* value
Age (years)	48 (43–53)	47 (42–52)	1.166 ^a^	0.244
Gender (male/female, n)	295/78	441/379	69.484 ^b^	0.000
Systolic blood pressure (mm Hg)	124.7 ± 10.3	118.3 ± 12.2	8.756	0.000
Diastolic blood pressure (mm Hg)	77.1 ± 7.5	72.3 ± 8.8	9.004	0.000
Body mass index (kg/m2)	26.3 ± 2.5	23.0 ± 2.6	20.010	0.000
Waist circumference (cm)	91.2 ± 7.7	81.1 ± 8.4	19.673	0.000
γ-glutamyltransferase (U/L)	40 (24–62)	18 (13–30)	13.931 ^a^	0.000
Triglyceride (mmol/L)	1.88 (1.32–2.73)	1.07 (0.80–1.50)	14.815 ^a^	0.000
Total cholesterol (mmol/L)	5.00 ± 0.93	4.64 ± 0.91	6.377	0.000
High-density lipoprotein (mmol/L)	1.04 ± 0.24	1.16 ± 0.29	-6.508	0.000
Low-density lipoprotein (mmol/L)	2.88 ± 0.68	2.68 ± 0.63	4.894	0.000
Very low density lipoprotein (mmol/L)	1.03 (0.71–1.36)	0.75 (0.48–1.00)	9.505 ^a^	0.000
Serum uric acid (μmol/L)	391.4 ± 83.7	318.2 ± 83.3	14.032	0.000
Fasting plasma glucose (mmol/L)	4.76 ± 0.47	4.55 ± 0.44	7.755	0.000
Glycosylated hemoglobin (%)	5.40 (5.20–5.70)	5.30 (5.03–5.50)	6.959 ^a^	0.000
eGFR (mL/min/1.73 m2)	107.0 ± 19.3	113.0 ± 23.4	-4.286	0.000

eGFR, estimated glomerular filtration rate; NAFLD, non-alcoholic fatty liver disease.

Data are expressed as mean (SD) or median (IQR).

^a^ Z value;

^b^ χ2 value.

### Association between eGFR levels and NAFLD related risk factors

Correlation analysis between eGFR and NAFLD related risk factors revealed an inverse correlation between eGFR levels and some NAFLD risk factors [DBP (*r* = -0.076, *P*<0.01), BMI (*r* = -0.156, *P*<0.001), WC (*r* = -0.204, *P*<0.001), GGT (*r* = -0.157, *P*<0.001), TG (*r* = -0.183, *P*<0.001), TC (*r* = -0.123, *P*<0.001), LDLc (*r* = -0.125, *P*<0.001), VLDLc (*r* = -0.125, *P*<0.001), SUA (*r* = -0.355, *P*<0.001), HbA1c (*r* = -0.082, *P*<0.01), and HDLc (*r* = 0.091, *P*<0.01)]. There was no significant correlation between eGFR levels and SBP, and same results were observed between eGFR levels and FPG (Both *P*>0.05, Table 2).

**Table 2:** Correlations between eGFR and NAFLD related risk factors.

	SBP	DBP	BMI	WC	GGT	TG	TC	HDL-C	LDL-C	VLDL-C	SUA	FPG	HbA1C
*r* value	-0.033	-0.076	-0.156	-0.204	-0.157	-0.183	-0.123	0.091	-0.125	-0.125	-0.355	0.019	-0.082
*P* value	0.259	0.009	0.000	0.000	0.000	0.000	0.000	0.002	0.000	0.000	0.000	0.518	0.005

SBP, Systolic blood pressure; DBP, Diastolic blood pressure; BMI, Body mass index; WC, Waist circumference; GGT, γ-glutamyltransferase; TG, Triglyceride; TC, Total cholesterol; HDLc, High-density lipoprotein; LDLc, Low-density lipoprotein; VLDLc, Very low density lipoprotein; SUA, Serum uric acid; FPG, Fasting plasma glucose; HbA1c, Glycosylated hemoglobin.

### Association of eGFR levels and NAFLD in different ages

To investigate the relationship between eGFR levels and NAFLD in different ages, all subjects were classified into five phases according to age: phase one (P1), age ≤40 years; phase two (P2), age 41–45 years; phase three (P3), age 46–50 years; phase four (P4), age 51–55 years; phase five (P5), age >55 years. Average eGFR levels of subjects with and without NAFLD were analyzed.

As shown in Table 3, average eGFR levels of subjects with NAFLD was lower than subjects without NAFLD in phase one, two and three (All *P*<0.05). However, there was no significant difference on average eGFR levels between NAFLD and non-NAFLD in phase four and five (Both *P*>0.05).

**Table 3:** Relationships between eGFR and NAFLD in different ages.

	No. of subjects	eGFR (mL/min/1.73 m2)	*T* value	*P* value
Age (year)	≤40			
With NAFLD (n)	86	108.6 ± 19.2	4.159	0.000
Without NAFLD (n)	226	122.0 ± 27.4		
Age (year)	41–45			
With NAFLD (n)	85	108.6 ± 20.3	2.717	0.007
Without NAFLD (n)	171	116.0 ± 20.1		
Age (year)	46–50			
With NAFLD (n)	72	108.9 ± 19.4	2.095	0.037
Without NAFLD (n)	125	117.8 ± 21.0		
Age (year)	51–55			
With NAFLD (n)	99	103.3 ± 17.9	1.135	0.257
Without NAFLD (n)	210	108.9 ± 18.5		
Age (year)	≥55			
With NAFLD (n)	64	101.9 ± 17.1	0.354	0.724
Without NAFLD (n)	148	102.9 ± 20.5		

**Fig 1:**
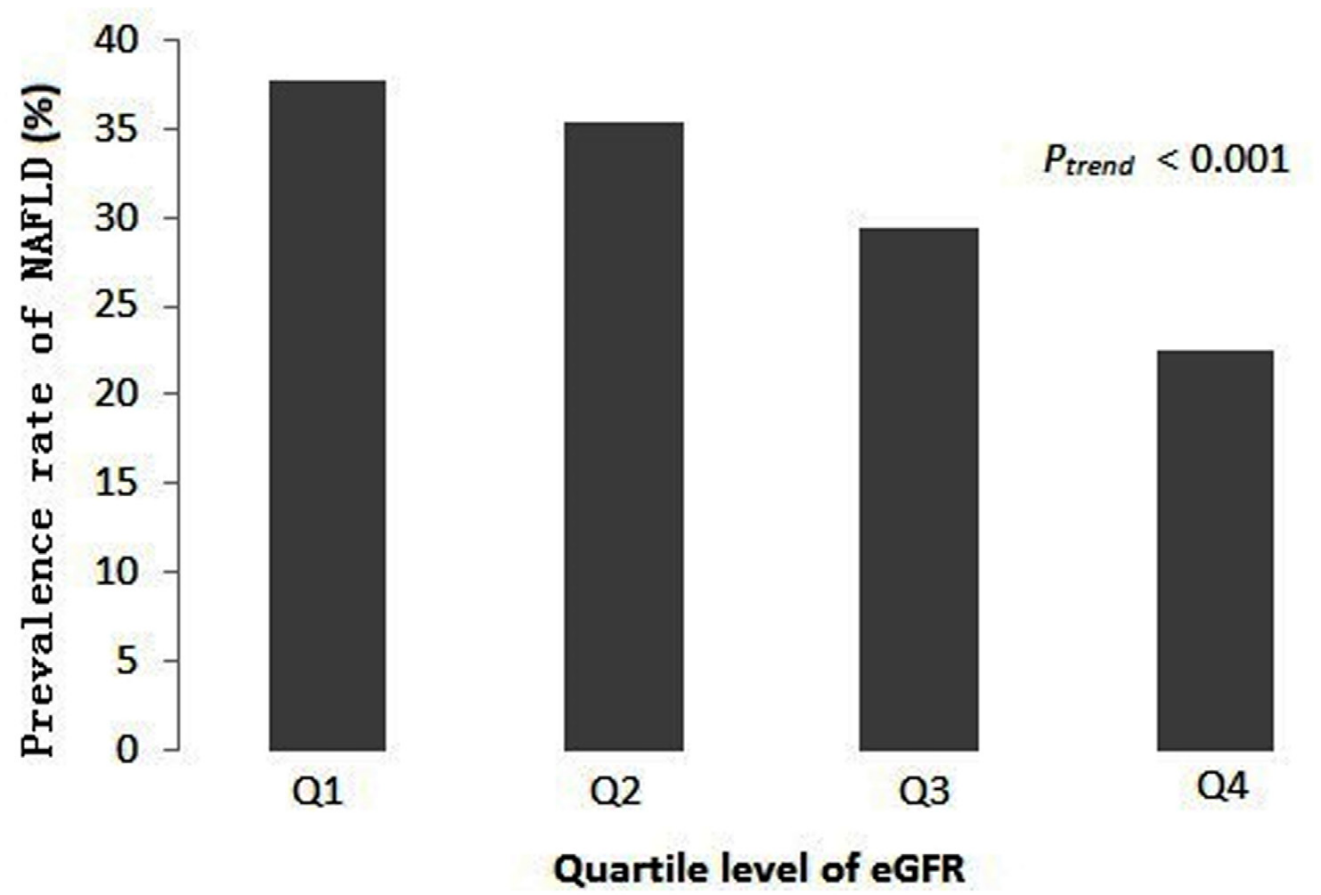
Comparison of relationships between eGFR and NAFLD in different age groups.

As shown in Fig 1, there were significant differences on average eGFR levels between NAFLD and non-NAFLD in the previous three phases of the five phases (All *P*<0.05). Moreover, there was no significant difference on the average eGFR level of NAFLD among the three phases (*P*>0.05). However, there was a significant difference on the average eGFR level of NAFLD between the previous three phases and the later two phases (*P*<0.05).

## Discussion

NAFLD is one of the most common chronic liver diseases, which is closely associated with metabolic syndrome; and its individual components include elevated blood pressure, dyslipidemia, high plasma glucose, obesity, and so on [2,20]. Renal dysfunction and injury is also a common health problem in modern society; and similar to NAFLD, CKD has also been linked to metabolic syndrome and its individual components [21,22]. Therefore, the link between NAFLD and CKD has been pushed to the forefront of research. A meta-analysis on the association of NAFLD and CKD has been conducted to determine whether the presence and severity of NAFLD is associated with the increased risk and severity of CKD. In our study, we further clarified the influence of NAFLD to kidney function in the early phase.

In this study, the comparison of variables between NAFLD and non-NAFLD subjects revealed a significant difference on average eGFR level, except for other well-known variables in an apparently healthy population. Targher G et al believed that fatty and inflamed liver produces pro-inflammatory factors which can further worsen kidney function [23]. Machado MV et al found that the presence and severity of liver inflammation correlated linearly with lower eGFR in their study [24].

Additionally, our data revealed that eGFR was significantly associated with most NAFLD risk factors including blood pressure, obesity parameters, liver enzyme, lipid level, uric acid, and HbA1c. A seven-year prospective cohort study performed by Wang Q *et al*. revealed a dose-response relationship between baseline blood pressure and eGFR level decline [25]. Kalaitzidis RG *et al*. believed that obesity was associated with increased risk of CKD and that a direct relationship exists between BMI and CKD risk. Moreover, central obesity measurements such as waist circumference could be a better predictor of CKD progression and mortality [14]. A US general population survey from 2001 to 2006 revealed a strong and independent relationship of increased serum GGT concentrations with CKD [26]. Hou X *et al*. thought that TC and TG were closely associated with mildly reduced eGFR levels in subjects with normal serum lipid levels [27]. A cohort study by Chou YC *et al*. revealed that elevated SUA levels independently predicted the risk of new-onset CKD [28]. A longitudinal observational study by Yokoyama H *et al*. demonstrated that a higher HbA1c baseline resulted in a more rapid GFR decline [29]. These studies suggest that NAFLD may not only be associated with renal dysfunction, but also be actively involved in its pathogenesis. There may be several explanations on the effects of NAFLD on eGFR levels such as disturbed tumor necrosis factor system, imbalance of renin-angiotensin-aldosterone system, insulin resistance, and chronic inflammation [14,23,30,31]. However, underlying mechanisms between the association of NAFLD and renal dysfunction remains unclear.

Age is an important risk factor for kidney disease; and eGFR is calculated by age, which is one of the important parameters. Thus, the older the age, the more the decline in eGFR level is affected. Additionally, age was also strongly associated with the development and progression of NAFLD [32]. Our data revealed that average eGFR levels of subjects with NAFLD was lower than controls when age was lower than 50 years, while there was no significant difference on average eGFR level between NAFLD and controls when age was more than 50 years. The fact that differences vanished after the age of 50, suggests that probably the importance the common risk factors for NAFLD and diminished eGFR is lost after a certain age. Certainly, the certain age needs to be confirmed in a follow-up study.

Nevertheless, there was a certain limitation in our study. Firstly, this study did not provide information on NAFLD disease severity duing to lack of hepatic pathology, it was not able to undestand the relationship between inflammation levels and eGFR levels. Secondly, this study only performed a cross-sectional analysis, and was not able to provide a cause and effect link or determine the underlying mechanism. In summary, our results revealed that eGFR levels significantly decreased in NAFLD compared to the control group, eGFR levels were significantly associated with related variables of NAFLD, and the effect of NAFLD on eGFR could be dependent on age. Certainly, further studies are needed to understand the potential mechanism of the association between NAFLD and eGFR levels through animal experiments and follow-up studies.
